# Identification and Expression Analysis of the Cytochrome P450 Genes in *Phyllotreta striolata* and CYP6TH1/CYP6TH2 in the Involvement of Pyridaben Tolerance

**DOI:** 10.3390/insects17010029

**Published:** 2025-12-24

**Authors:** Yongqin Zhu, Zhongting Liu, Wenyong Mai, Xinhua Pu, Haoyue Mo, Benshui Shu, Zhongzhen Wu

**Affiliations:** 1 Guangzhou City Key Laboratory of Subtropical Fruit Tree Outbreak Control, Zhongkai University of Agriculture and Engineering, Guangzhou 510225, China; 2 National Key Laboratory of Green Pesticide, Department of Pesticide Science, College of Plant Protection, South China Agricultural University, Guangzhou 510642, China

**Keywords:** cytochrome P450, *Phyllotreta striolata*, pyridaben, insecticide tolerance, RNA interference

## Abstract

The striped flea beetle, *Phyllotreta striolata*, is a worldwide pest that causes severe damage to cruciferous crops. Pyridaben, an inhibitor of mitochondrial complex I, has been widely used for the control of *P. striolata*. Identifying key genes involved in pyridaben tolerance is vital for establishing effective resistance management strategies. A total of 94 full-length cytochrome P450 (CYP) genes were identified in *P. striolata*. The identified genes were classified into four clans (mitochondrial, CYP2, CYP3, and CYP4), 23 families, and 57 subfamilies. Transcriptomic analysis across developmental stages and tissues revealed distinct expression patterns were seen in these P450 genes. Pyridaben exposure significantly upregulated the expression of *CYP6TH1* and *CYP6TH2*. Tissue-specific expression profiling indicated that *CYP6TH1* and *CYP6TH2* were highly expressed in the head and fat body. RNA interference-mediated knockdown of these genes significantly increased the susceptibility of *P. striolata* to pyridaben. Molecular docking confirmed stable binding between pyridaben and both CYP proteins, with binding free energies of −7.73 and −7.57 kcal/mol for CYP6TH1 and CYP6TH2, respectively. This study demonstrates that CYP6TH1 and CYP6TH2 play critical roles in pyridaben tolerance in *P. striolata*, which highlights promising genetic targets for future resistance management strategies.

## Simple Summary

Pyridaben is extensively utilized to manage *Phyllotreta striolata*; nevertheless, the emergence of resistance from continuous use has created difficulties for its efficient control. At present, research on the significance of Cytochrome P450 genes in imparting pesticide resistance in *Phyllotreta striolata* is scarce. This research discovered 94 cytochrome P450 genes in *Phyllotreta striolata*, examined their spatiotemporal expression patterns, and demonstrated that *CYP6TH1* and *CYP6TH2* are crucial for pyridaben tolerance.

## 1. Introduction

The striped flea beetle, *Phyllotreta striolata* (Fabricius) (Coleoptera: Chrysomelidae), is a destructive pest of cruciferous vegetables [1]. Both larvae and adults cause significant damage to plants: adults feed on the leaf mesophyll, producing characteristic “shot-hole” symptoms, and in severe cases, may consume the entire mesophyll layer. Larvae can damage main roots and root hairs, leading to plant wilting and eventual death [2]. Chemical insecticides such as fipronil, chlorpyrifos, phoxim, imidacloprid, and acetamiprid are widely used to control *P. striolata* [3,4,5]. However, intensive and repeated application has led to the emergence of resistant strains in recent years [6].

Pyridaben, a pyridazinone derivative that inhibits mitochondrial complex I in the electron transport chain [7], is another extensively used insecticide. As a miticide, pyridaben is also extensively used to control *P. striolata* [8]. For example, field trials have shown that 15% pyridaben EC is among the most effective insecticides recommended for managing this pest [9]. Additionally, combinations such as pyridaben–acetamiprid WP show significant synergy [10]. At the same time, many pest species around the world have been found to be very resistant to pyridaben. For instance, *Tetranychus urticae* [11] and *Panonychus citri* [12] have shown strong resistance to pyridaben in fields. In Yunnan Province, China, field populations of *Tetranychus cinnabarinus* exhibited resistance ratios to pyridaben ranging from 1.50- to 4.68-fold [13]. High levels of resistance of *P. striolata* to pyridaben have also been observed in Guangdong Province, where pyridaben has been applied extensively for insect pest management [6]. Thus, a detailed understanding of how P. striolata responds to pyridaben at the molecular level is essential.

Cytochrome P450 monooxygenases (P450s or CYPs), one of the largest enzyme superfamilies across living organisms, play crucial roles in metabolizing both endogenous and exogenous compounds, including steroids, fatty acids, and insecticides. The constitutive overexpression of specific P450 genes is a well-documented mechanism underlying insecticide resistance [14]. For example, *CYP6DB3* is upregulated in thiamethoxam- and imidacloprid-resistant strains of *Bemisia tabaci* [15]. Elevated P450 activities and overexpression of *CYP6BG1* have been linked to chlorantraniliprole resistance in *Plutella xylostella* [16]. In *Spodoptera exigua*, multiple CYP6AE genes, particularly *CYP6AE70*, are overexpressed and associated with cross-resistance to chlorpyrifos, cypermethrin, and deltamethrin [17]. Additionally, pyridaben exposure upregulates *CYP4CF1* and *CYP4CL2* in *Panonychus citri* [18]. However, the involvement of P450 genes in insecticide resistance has been poorly investigated in *P. striolata*.

In this study, we identified 94 full-length P450 genes and analyzed their expression patterns across different developmental stages and tissues. We further investigated the response of these P450 genes to sublethal pyridaben exposure (LC_10_ and LC_50_). Knockdown of these two genes via RNA interference (RNAi) increased susceptibility of *P. striolata* to pyridaben. The potential interactions between CYP6TH1/CYP6TH2 and pyridaben were analyzed through molecular docking experiments. Our findings offer new insights into the molecular mechanisms of P450-mediated pyridaben tolerance in *P. striolata*.

## 2. Materials and Methods

### 2.1. Insect Rearing

The *P. striolata* population used in this study was a laboratory colony originally collected from a crucifer crop field in Fujian province, China. Since 2014, the strain has been maintained on mustard plants (*Brassica juncea* cv. Bau-Sin) in a greenhouse at Zhongkai University of Agriculture and Engineering without exposure to insecticides. Rearing conditions were as follows: temperature 21–26 °C, relative humidity 60–80%, and soil humidity 95%. Natural daylight was used as the light source in the greenhouse.

### 2.2. Toxicological Bioassay

The susceptibility of *P. striolata* adults to pyridaben (analytical standard, Sigma-Aldrich, St. Louis, MO, USA) was determined using the leaf-dip bioassay method. A stock solution of pyridaben was prepared in acetone and diluted to various concentrations for assays. Fresh, uniform radish leaf disks (diameter: 2 cm) were dipped in the corresponding insecticide solutions for 10 s, with control leaf disks dipped in acetone only. The treated leaf disks were air-dried for approximately 2 h to allow the solvent to evaporate completely. For each concentration, 60 actively moving adults (3–5 days old; 20 adults for each replicate, and three replicates were prepared) were starved for 2 h and then introduced into a Petri dish (9 cm diameter) containing the treated leaf disk. The treated leaf disks were replaced daily to ensure fresh food supply and continuous exposure. A moistened cotton ball was placed in the dish to prevent desiccation. Mortality was assessed after 48 h of exposure. Adults were considered dead if they did not move when prodded with a fine brush. The experiment was repeated three times independently. Probit analysis (SPSS version 19.0) was used to calculate the LC_10_ and LC_50_ values along with their 95% confidence intervals.

### 2.3. Sample Preparation for RNA-Seq

Transcriptomic analyses were performed on three sets of samples from *P. striolata*: (1) Different developmental stages: eggs, 3rd instar larvae, pupae, and 3-day-old adults. (2) Different tissues of 3-day-old adults: dissected heads, ovaries, midguts, Malpighian tubules, and fat bodies (male/female = 1:1). (3) whole bodies of 3-day-old adults after a 48 h treatment with sublethal doses (LC_10_ and LC_50_) of pyridaben. The LC_10_ dose was employed to simulate a mild stress environment for capturing early responsive genes, while the LC_50_ dose was selected to identify key tolerance genes under high selection pressure, thereby avoiding the physiological collapse associated with higher lethal concentrations. Survivors were collected for RNA extraction. Samples from the developmental stages and tissues included three biological replicates, while the pyridaben-treated groups included two biological replicates, respectively. Each biological replicate comprised 50 individuals (or pooled tissues from 50 individuals), which were immediately frozen in liquid nitrogen and stored at −80 °C until RNA extraction.

### 2.4. RNA Extraction, Library Construction, and Sequencing

Total RNA was extracted from each sample using TRIzol^®^ Reagent (Invitrogen, Carlsbad, CA, USA) following the manufacturer’s protocol. RNA integrity and concentration were assessed using an Agilent 2100 Bioanalyzer (Agilent Technologies, Santa Clara, CA, USA) and a NanoDrop spectrophotometer (Thermo Fisher Scientific, Waltham, MA, USA). Only samples with an RNA Integrity Number (RIN) > 8.0 were used for subsequent library construction. Sequencing libraries were generated using a NEBNext^®^ Ultra™ RNA Library Prep Kit from Illumina^®^ (New England Biolabs, Ipswich, MA, USA) following the manufacturer’s recommendations. The libraries were sequenced on an Illumina NovaSeq 6000 platform (Illumina, San Diego, CA, USA), generating paired-end reads of approximately 150 bp in length.

### 2.5. Data Processing and Differential Expression Analysis

Raw sequence reads were processed through a stringent quality control pipeline. Adapter sequences and low-quality reads were removed using Trimmomatic. The clean reads were de novo assembled into transcripts using Trinity software (version 2.8.5). The assembled unigenes were functionally annotated against major databases, including NR, NT, Pfam, KOG/COG, Swiss-Prot, KEGG, and GO. Gene expression levels were estimated by calculating TPM (Transcripts Per Million) values. Differential expression analysis was conducted using the DESeq2 R package (version 1.26.0) with raw read counts as input. Genes with an adjusted *p*-value (padj) < 0.05 and |log_2_(FoldChange)| > 1 were assigned as differentially expressed genes (DEGs).

### 2.6. P450 Gene Identification, Phylogeny, and Expression Profiling

Putative P450 genes in *P. striolata* were identified by performing a local BLAST search against its genome assembly, which was retrieved from NCBI (https://www.ncbi.nlm.nih.gov/datasets/genome/GCA_918026865.1/ (accessed on 1 December 2025)). The search used known insect P450 protein sequences from *Tribolium castaneum* as queries. Candidate genes were further verified by detecting the conserved P450 domain (PF00067) using InterProScan and the Pfam database. The coding completeness of the candidate *P. striolata* P450s was assessed before obtaining official designations based on the criteria established by the P450 Nomenclature Committee [19].

The deduced amino acid sequences of the identified *P. striolata* P450s were aligned with P450s from other model insect species using MAFFT (version 7.1). A phylogenetic tree was reconstructed using the neighborhood-joining method based on FastTree. The robustness of the tree was evaluated with 1000 bootstrap replicates. The resulting tree was visualized and annotated using iTOL (https://itol.embl.de/ (accessed on 20 December 2025)).

To further determine the expression levels of the P450 genes identified in the *P. striolata* genome, the clean reads were then pseudoaligned to the transcript sequences from the reference genome using Kallisto, and gene expression levels were quantified as TPM (Transcripts Per Million). Heatmaps were generated using the TBtools-II software (version 2.382) to visualize the expression profiles [20].

### 2.7. qRT-PCR Validation

To validate the transcriptome sequencing results, the expression levels of key P450 genes identified as differentially expressed were analyzed using qRT-PCR. Total RNA was extracted from the whole bodies of surviving adults (dead individuals were excluded) collected after 48 h of exposure (consistent with the bioassay duration) to control and pyridaben-treated (LC_10_ and LC_50_) conditions. First-strand cDNA was synthesized from 1 µg of DNase I-treated total RNA using the PrimeScript RT reagent Kit with gDNA Eraser (Takara Bio Inc., Kusatsu, Shiga, Japan).

Gene-specific primers were designed using Primer Premier 5.0 software, with amplicon lengths ranging from 80 to 200 bp. The *β-Actin* gene was selected as an internal reference based on its stable expression in the transcriptome data. qRT-PCR was performed in triplicate for each sample using TB Green^®^ Premix Ex Taq™ II (Takara Bio Inc., Kusatsu, Shiga, Japan) on a QuantStudio 5 Real-Time PCR System (Applied Biosystems, Foster City, CA, USA). The reaction protocol consisted of an initial denaturation at 95 °C for 30 s, followed by 40 cycles of 95 °C for 5 s and 60 °C for 34 s. A melt curve analysis was performed at the end of each run to confirm the specificity of amplification. The relative expression levels of target genes were calculated using the 2^(−ΔΔCt)^ method.

### 2.8. RNA Interference and Toxicity Bioassay

Based on their expression profiles, *CYP6TH1* and *CYP6TH2* were selected for RNA interference (RNAi). Specific double-stranded RNA (dsRNA) primers targeting *CYP6TH1*, *CYP6TH2*, and *GFP* (as a control) were designed using E-RNAi (https://e-rnai.dkfz.de/signaling/e-rnai3/ (accessed on 10 April 2024)), each incorporating a T7 promoter sequence at the 5′ end (see Supplementary Table S3 for primer details). These primers were amplified from *P. striolata* cDNA samples or from a plasmid harboring the *GFP* gene. PCR products were confirmed by sequencing and then used as templates for in vitro transcription using the T7 RiboMAX™ Express RNAi System (Promega, Madison, WI, USA), following the manufacturer’s instructions. The synthesized dsRNA (3000–5000 ng/μL) was quantified using a NanoDrop 2000 spectrophotometer (Thermo Fisher Scientific, Waltham, MA, USA), and its integrity was assessed via agarose gel electrophoresis. dsRNA samples were aliquoted and stored at −80 °C until further use.

Microinjection of dsRNA into insects was performed. Approximately 200 ng of either ds*GFP* (control) or gene-specific dsRNA was injected into each *P. striolata* adult. RNAi efficiency was assessed using qPCR at 24, 48, and 72 h post-injection, with cDNA synthesized from isolated total RNA. The impact of dsRNA on target gene expression was analyzed using qPCR with ds*GFP*-injected insects as the negative control. Separately, adults treated with dsRNA were transferred to fresh *B. juncea* leaves that had been treated with LC_10_ or LC_50_ doses of pyridaben and allowed to air-dry. Mortality was recorded over a 72 h period. Each RNAi-treated group consisted of 20 adults, and the entire bioassay was independently replicated three times.

### 2.9. Molecular Docking

The three-dimensional (3D) structures of CYP6TH1 and CYP6TH2 proteins were predicted using AlphaFold3 (https://alphafold.com). The stereochemical quality and structural validity of the predicted models were evaluated using the SAVES v6.1 server (https://saves.mbi.ucla.edu/ (accessed on 15 August 2025)). The 3D structure of pyridaben was obtained from the PubChem database (https://pubchem.ncbi.nlm.nih.gov/ (accessed on 15 August 2025)). The proteins and ligand were first prepared for docking using AutoDock Tools v1.5.4 [21]. Molecular docking simulations were then performed with AutoDockTools-1.5.7 to explore the binding interactions between the proteins (CYP6TH1 and CYP6TH2) and pyridaben. The resulting complexes were visualized and analyzed using PyMOL (version 3.0.3). The interactions between pyridaben and the active sites of CYP6TH1 and CYP6TH2 were visualized using Discovery Studio (version 4.5).

### 2.10. Statistical Analysis

SPSS version 16.0 (SPSS, Inc., Chicago, IL, USA) was applied to perform statistical analyses. Probit analysis was used to calculate the LC_10_ and LC_50_ values along with their 95% confidence intervals. Student’s *t*-test was used to evaluate statistically significant differences between two samples. Mortality data among adult insects were analyzed using one-way ANOVA at *p* < 0.05.

## 3. Results

### 3.1. Identification and Phylogenetic Analysis of CYP Genes

**Figure 1:**
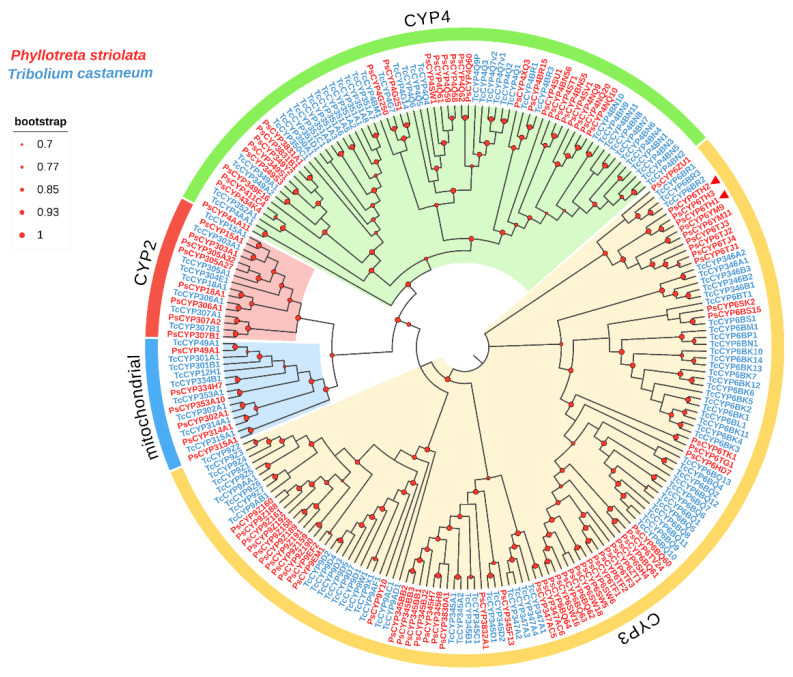
Phylogenetic analysis of CYP genes from *P. striolata* and *T. castaneum* constructed using the maximum-likelihood method. Clades are colored according to CYP clan: CYP2 (pink), CYP3 (yellow), CYP4 (green), and mitochondrial (blue). Branch support values (circles at nodes) were estimated using an approximate likelihood ratio test (see scale, top left). Red triangles indicate the target genes.

Based on the *P. striolata* genome, 94 full-length *PsCYP* open reading frames (ORFs) were identified through keyword search and domain analysis (Table S1). The 94 full-length *PsCYPs* were officially named following the criteria established by the P450 Nomenclature Committee (Dr. D. Nelson, personal communication). According to the standard classification system, these 94 *PsCYPs* were grouped into four major clans: mitochondrial (6 genes), CYP2 (8 genes), CYP3 (53 genes), and CYP4 (27 genes). These clans were further subdivided into 23 families and 57 subfamilies (Table S2). A neighborhood-joining phylogenetic tree was constructed to infer the orthologous relationships of P450 sequences between *T. castaneum* and *P. striolata* (Figure 1). The tree resolved the four major P450 clans: CYP2, CYP3, CYP4, and mitochondrial. The CYP2 and mitochondrial clans comprised highly conserved members with well-established functions, whereas the CYP3 and CYP4 clans showed greater diversification, with many genes having unknown functions.

Among the conserved clans, four genes, three from the mitochondrial clan and one from CYP2, were identified as Halloween genes (*CYP302A1*, *CYP306A1*, *CYP307A2*, *CYP314A1*, and *CYP315A1*), which are known to encode enzymes involved in the synthesis of the molting hormone 20-hydroxyecdysone, as previously reported in *D. melanogaster* [22]. Another gene from the CYP2 clan, *CYP18A1*, is likely implicated in 20-hydroxyecdysone degradation in insects. Within the CYP3 and CYP4 clans, four and three distinct clusters of *P. striolata* CYPs were identified, respectively. Members of these clans are typically associated with the metabolism of pesticides, plant secondary metabolites, odorants, and pheromones. The expansion of CYP3 and CYP4 clans in *P. striolata* may contribute to its high capacity for detoxification and insecticide resistance.

### 3.2. Tissue and Stage Expression Pattern

**Figure 2:**
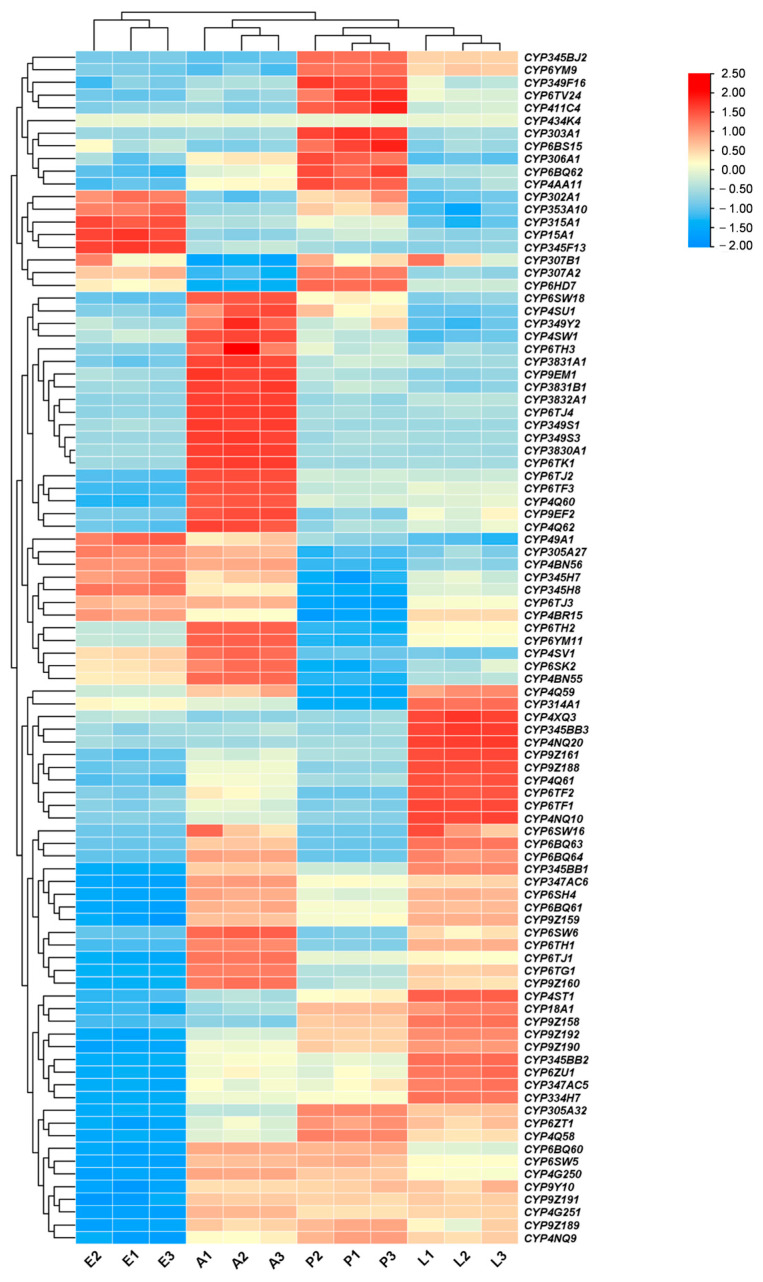
Expression patterns of CYP genes across different developmental stages of *P. striolata* based on TPM values. Samples include Eggs (E), Adults (A), Pupae (P), and Larvae (L) (indicated below the heatmap). Color intensity reflects expression levels, with red indicating upregulation and blue indicating downregulation.

To gain insight into the potential functions of CYP genes in *P. striolata*, we analyzed their expression patterns across different developmental stages and adult tissues using TPM values. Developmental stage expression analysis clustered the 94 CYP genes into distinct clades (Figure 2). Eggs highly expressed a group of 12 genes, including *CYP302A1*, *CYP353A10*, and *CYP315A1*. Another 23 genes, such as *CYP6SW18*, *CYP4US1*, *CYP349Y2*, *CYP4SW1*, *CYP6TH3*, and *CYP3831A1*, showed elevated expression in adult stages. Additionally, 20 genes were predominantly expressed in larvae, while 10 genes exhibited peak expression in pupae.

**Figure 3:**
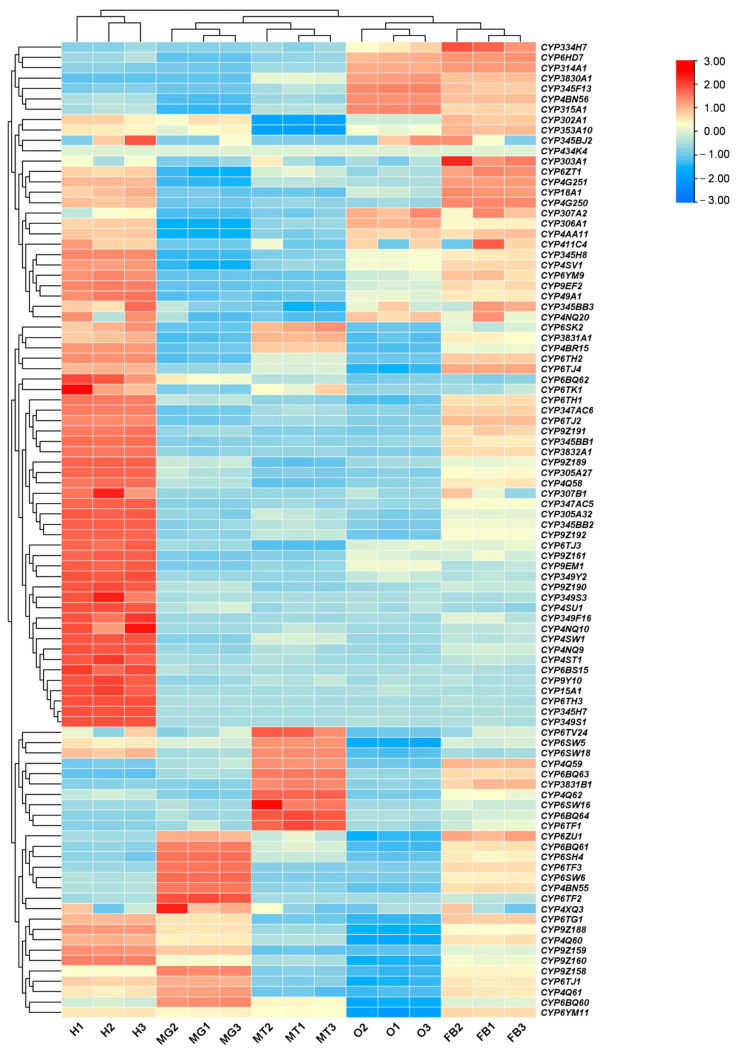
Tissue-specific expression profiles of CYP genes in *P. striolata* based on TPM values. Tissues analyzed include Heads (H), Midguts (MG), Malpighian tubules (MT), Ovaries (O), and Fat bodies (FB) (shown below the heatmap). Red and blue shades represent upregulation and downregulation, respectively.

Tissue-specific expression analysis revealed diverse patterns of CYP gene expression in various tissues (Figure 3). A group of 12 genes showed relatively high expression in the midgut, while another 10 genes were highly expressed in the Malpighian tubules. Thirty-nine genes exhibited elevated expression in the head. Another 5 genes were predominantly expressed in fat bodies. These results reveal that CYP genes are differently expressed in specific tissues of *P. striolata*, suggesting functional specializations connected to their expression sites. CYPs abundantly expressed in the midgut, Malpighian tubules and fat body are likely involved in the detoxification and metabolism of pesticides or plant secondary chemicals. Those expressed in the head may play roles in neurological or sensory processes.

### 3.3. Impact of Pyridaben on Insect Survival and P450 Gene Expression

As summarized in Table S4, pyridaben exhibited relatively low insecticidal activity against adult *P. striolata*. After 24 h of exposure, mortality rates were 1.19 ± 1.19%, 18.75 ± 6.25%, 29.27 ± 8.00%, 43.48 ± 9.32%, and 54.67 ± 5.81% at concentrations of 100, 125, 166.7, 250, and 500 μg/mL, respectively. Based on the mortality data, the probit regression equation was fitted as y = 2.8504x − 2.2559, where y represents the probability of mortality and x denotes the logarithm of concentrations. The LC_50_ and LC_10_ values were 351.2 μg/mL and 180.3 μg/mL, respectively.

**Figure 4:**
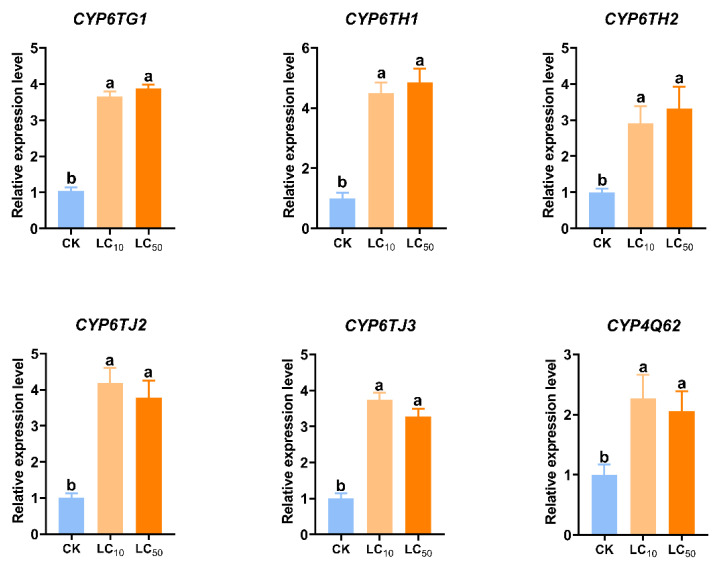
Expression levels of CYP genes in *P. striolata* after pyridaben exposure, as validated by RT-qPCR. Data represent mean ± SE (*n* = 3). Different lowercase letters above bars indicate significant differences among groups (*p* < 0.05, one-way ANOVA). CK: control group; LC_10_: group treated with LC_10_ pyridaben; LC_50_: group treated with LC_50_ pyridaben.

The expression levels of CYP genes in adults following 24 h exposure to LC_10_ and LC_50_ pyridaben treatments are shown in Figure S1. Among the identified CYP genes, *CYP6TG1*, *CYP6TH1*, *CYP6TH2*, *CYP6TJ2*, *CYP6TJ3*, and *CYP4Q62*, were upregulated under both doses. Four of them, including *CYP6TH1*, *CYP6TH2*, *CYP6TJ2*, and *CYP6TJ3*, which are from the same clade, showed more than four-fold upregulation. The upregulation was further validated by RT-qPCR (Figure 4).

### 3.4. Knockdown of CYP6TH1 and CYP6TH2 Reduces Survival of P. striolata to Pyridaben Treatments

**Figure 5:**
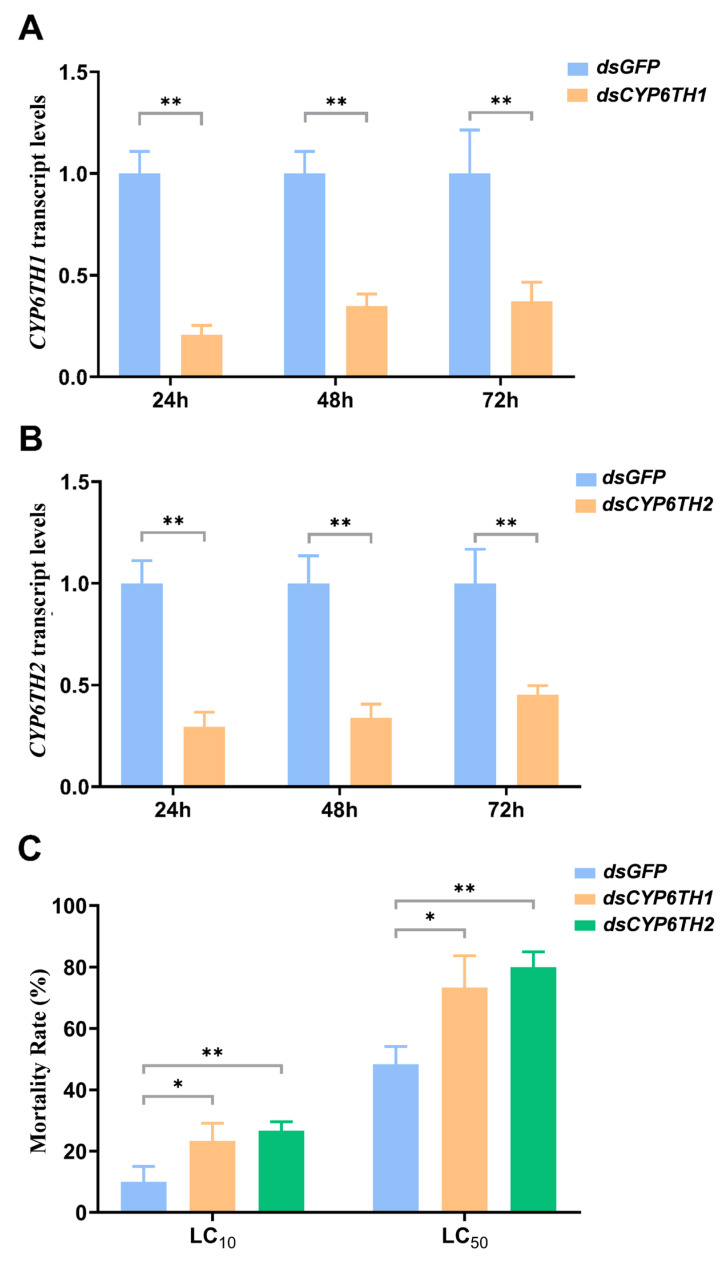
RNA interference-mediated knockdown of *CYP6TH1* and *CYP6TH2.* (**A**,**B**) Knockdown efficiency of *CYP6TH1* and *CYP6TH2* after dsRNA injection. (**C**) Changes in pyridaben susceptibility of *P. striolata* adults following RNAi. Data represent mean ± SE (*n* = 3). Significance is indicated as * *p* < 0.05, and ** *p* < 0.01 (Unpaired *t*-test).

To investigate the role of *CYP6TH1* and *CYP6TH2* in pyridaben tolerance, RNAi was employed to knock down their expression in *P. striolata* adults (Figure 5). Compared to the ds*GFP*-treated controls, ds*CYP6TH1* resulted in significant reductions in *CYP6TH1* expression, with 79.36%, 65.14%, and 62.92% reduction in transcript levels at 24, 48, and 72 h post dsRNA-injection, respectively (Figure 5A; *p* < 0.05, Unpaired *t*-test). ds*CYP6TH2* injection also significantly suppressed *CYP6TH2* expression, with 70.62%, 66.23%, and 54.71% reduction at the above-mentioned time points (Figure 5B; *p* < 0.05 Unpaired *t*-test). Correlated with gene silencing, susceptibility to pyridaben was markedly enhanced: mortality under LC_10_ exposure increased by 12.33% and 16.33% in ds*CYP6TH1*- and ds*CYP6TH2*-treated beetles, respectively. Mortality rose even higher following LC_50_ exposure, with 25.00% and 31.67% increases in insect death rates, respectively (Figure 5C).

### 3.5. Predicted Binding of CYP6TH1 and CYP6TH2 with Pyridaben

**Figure 6:**
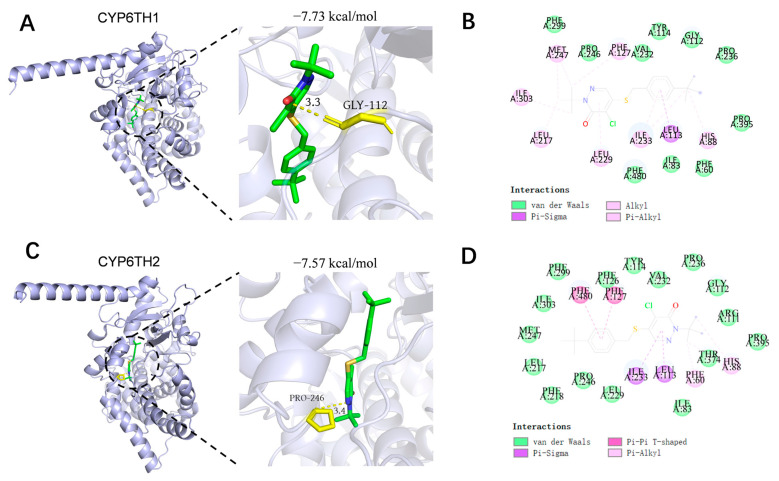
Molecular docking prediction model between CYP6TH1 and CYP6TH2 of *P. striolata* and pyridaben. (**A**,**C**) Three-dimensional visual analysis for the binding mode of pyridaben to CYP6TH1 and CYP6TH2. The green structure represents pyridaben, and the yellow structures represent the interacting amino acid residues. (**B**,**D**) Interactions at the active sites of the representative conformation of pyridaben to CYP6TH1 and CYP6TH2.

To gain more profound insights into the potential interactions between pyridaben and CYP6TH1/CYP6TH2, molecular docking simulations were performed. The results revealed that the binding of pyridaben to CYP6TH1 was primarily stabilized by van der Waals forces and hydrophobic interactions (Figure 6A,B). A total of 16 amino acid residues of CYP6TH1 (Ile83, Arg111, Gly112, Tyr114, Phe126, Leu217, Phe218, Leu229, Val232, Pro236, Pro246, Met247, Phe299, Ile303, Thr374, and Pro395) were found to interact with pyridaben via van der Waals forces. Additionally, 6 residues (Phe60, His88, Leu113, Phe127, Ile233, and Phe480) formed hydrophobic interactions with the insecticide (Figure 6B). Similarly, van der Waals forces and hydrophobic interactions were also identified as the main driving forces for the binding of pyridaben to CYP6TH2 (Figure 6C,D). For instance, 10 residues (Phe60, Ile83, Gly112, Tyr114, Val232, Pro236, Pro246, Phe299, Pro395, and Phe480) participated in van der Waals interactions with pyridaben. Furthermore, the binding free energies of CYP6TH1 and CYP6TH2 with pyridaben were −7.73 kcal/mol and −7.57 kcal/mol, respectively, indicating strong binding affinity between CYP6TH1 and pyridaben and between CYP6TH2 and pyridaben.

## 4. Discussion

Chemical insecticides remain one of the most direct and effective approaches for pest management. However, insects have developed diverse resistance mechanisms in response to prolonged selection pressure. These include the overexpression of detoxification enzymes and structural mutations that enhance catalytic efficiency through amino acid substitutions [23,24,25]. Key detoxifying enzymes involved in insecticide metabolism are cytochrome P450 monooxygenases (P450s), glutathione S-transferases (GSTs), and carboxylesterases (CarEs) [26]. Among these, P450s are particularly versatile, catalyzing oxidation–reduction reactions critical for numerous physiological and detoxification processes. The advent of high-throughput sequencing has greatly accelerated the identification of P450 genes, including in non-model species lacking reference genomes. To date, P450s have been characterized in many major insect pest species; for instance, 77 P450-encoding genes have been identified in *Chilo suppressalis*, 58 in *Bemisia tabaci* MED, 103 in *Tenebrio molitor*, and 71 in *Bactrocera dorsalis* [27,28,29,30]. In this study, we identified 94 full-length *CYP* genes in *P. striolata* for the first time. These were classified into four clans: CYP2, CYP3, CYP4, and mitochondria. The genes were further subdivided into 23 families and 57 subfamilies. Considerable interspecific variations in *CYP* gene numbers were observed, consistent with the high evolutionary dynamism of P450-encoding genes in adaptation to xenobiotic metabolism. Particularly, genes in the CYP3 clan undergo frequent gene duplication and loss along with point mutations [31]. Gene duplication and nucleotide substitutions coupled with positive selection led to functional divergence, driving P450-encoding gene diversification. This study identified expansions in specific CYP subfamilies in P. striolata. Four distinct clades were identified within the CYP3 clan. For example, the CYP6TJ and CYP6TH subfamilies formed one clade, while the CYP6BQ, CYP6SW, CYP6TF, CYP6SH, CYP6TG, CYP6TK, and CYP6HD subfamilies constituted another clade. These expanded clades may significantly enhance metabolic resistance to specific insecticides or plant secondary compounds [28,31,32].

Despite the expansion and diversity of some P450 clades, many other *CYP* genes are evolutionarily conserved across insect species. The conserved genes include the Halloween genes (*CYP302A1*, *CYP306A1*, *CYP307A2*, *CYP314A1*, and *CYP315A1*) and the *CYP18A1* gene. The conserved P450-encoding genes are usually essential for biosynthesis and inactivation of the molting hormone 20-hydroxyecdysone (20E) [33,34]. In our study, five Halloween genes (*CYP302A1*, *CYP306A1*, *CYP307A2*, *CYP314A1*, and *CYP315A1*) and the *CYP18A1* gene were identified in *P. striolata*, suggesting their conserved roles in ecdysteroid regulation. In addition to these well-known conserved P450-encoding genes, three other genes (*CYP305A1*, *CYP49A1*, and *CYP353A1*) were also found highly conserved. Of these, *CYP305A1* and *CYP49A1* have been reported to play a role in insect resistance to chlorantraniliprole, possibly through enhanced detoxification [35].

Spatiotemporal expression profiling revealed that most P450-encoding genes in *P. striolata*, particularly those in the CYP3 and CYP4 clans, were highly expressed in adults. This adult-biased expression may reflect increased metabolic demands and direct exposure to insecticides, necessitating robust detoxification capacity. In contrast, earlier developmental stages (larvae and pupae) are more sheltered within the soil matrix, reducing exposure to xenobiotics [36]. Halloween genes, which regulate ecdysteroid synthesis, were predominantly expressed in egg, larval, or pupal stages—a pattern consistent with other insects. For example, in *B. dorsalis*, *CYP302A1* is highly expressed in eggs and contributes to embryonic cuticle formation [37]. Tissue-specific expression also aligned with physiological roles: the midgut, a primary site for digestion and xenobiotic defense [38,39], showed specific expression of *CYP49A1* in *Spodoptera frugiperda*, where its upregulation correlates with chlorantraniliprole resistance [35]. Three Halloween genes, including *CYP314A1*, *CYP315A1*, and *CYP306A1*, are highly expressed in the ovary, consistent with their role in adult ecdysteroid synthesis following the degeneration of the prothoracic gland [40].

Upregulation of detoxification genes in response to xenobiotics is a well-documented resistance mechanism [41,42,43,44,45]. For example, in *Helicoverpa armigera*, *CYP9A14* and *CYP6AE11* are induced by multiple insecticides and contribute to resistance [42]. Similarly, in *Bradysia odoriphaga*, lambda-cyhalothrin and imidacloprid induce *CYP6QE1* and *CYP6FV21* [46], and in *Locusta migratoria*, *CYP6FE1*, *CYP6FF1*, and *CYP6FG1* are associated with deltamethrin detoxification and resistance [47]. In this study, exposure of *P. striolata* to pyridaben resulted in significant upregulation of six P450-encoding genes, five of which belong to the CYP3 clan. To date, most P450s known to metabolize pesticides and xenobiotics have been identified within the CYP6 and CYP9 families of the CYP3 clan. Mounting evidence indicates that CYP6 family genes play important roles in insecticide resistance. For instance, *CYP6EM1* confers thiamethoxam resistance in *B. tabaci* [48], *CYP6CX4* contributes to flupyradifurone resistance [49], and *CYP6AQ83* enhances flonicamid tolerance in *Solenopsis invicta* [50]. Here, RNA interference targeting either *CYP6TH1* or *CYP6TH2* increased *P. striolata* susceptibility to pyridaben, suggesting their roles in pyridaben resistance. Although RNAi confirmed the involvement of CYP6TH1 and CYP6TH2 in pyridaben tolerance, they are likely not the sole contributors. Our data showed that other P450 genes, such as *CYP6TG1*, *CYP6TJ2*, and *CYP6TJ3*, were also upregulated upon exposure. Insecticide resistance is typically a complex trait involving functional redundancy or synergism among multiple enzymes. Therefore, the observed tolerance is likely the collective result of a broader P450 network. Molecular docking has been widely used to predict the binding affinity between detoxification enzymes and xenobiotics, especially in insecticide resistance studies [51,52,53]. Our docking results showed CYP6TH1 and CYP6TH2 have a strong binding affinity for pyridaben.

## 5. Conclusions

In summary, this study identified 94 full-length cytochrome P450-encoding genes in *P. striolata*. We characterized their spatiotemporal expression patterns and demonstrated that pyridaben upregulates *CYP6TH1* and *CYP6TH2*. RNAi assays provide initial evidence that *CYP6TH1* and *CYP6TH2* contribute to pyridaben tolerance. Molecular docking suggests stable binding of pyridaben to both CYP6TH1 and CYP6TH2 proteins. These findings underscore the importance of CYP6 subfamilies in insecticide tolerance and mark the first investigation into the role of the CYP6TH subfamily in pyridaben tolerance, as well as the first mechanistic insights into pyridaben tolerance in *P. striolata*.

## Data Availability

Data will be made available on request.

## Supplementary Materials

The following supporting information can be downloaded at: https://www.mdpi.com/article/10.3390/insects17010029/s1, Table S1: Characteristics of CYP genes of *P. striolata*. Table S2: Number of *P. striolata* full-length CYP families, subfamilies, and genes in each insect CYP clan. Table S3: Primers used in this study. Table S4: The mortality statistics for *P. striolata* under various pyridaben treatments. Figure S1: Expression patterns of CYP genes in *P. striolata* adults under pyridaben treatment for 24 h at LC_50_ and LC_10_ concentrations, based on FPKM values. Treatments include a control group and groups exposed to LC_10_ or LC_50_ pyridaben (indicated below the heatmap). Red and white colors denote upregulation and downregulation, respectively.
