# Supplementary material for: Identification and Expression Analysis of the Cytochrome P450 Genes in Phyllotreta striolata and CYP6TH1/CYP6TH2 in the Involvement of Pyridaben Tolerance
Source: Insects. 2025 Dec 24;17(1):29. doi: 10.3390/insects17010029 (PMC12842589; doi:10.3390/insects17010029)
Supplement: Supplementary file 1 [file insects-17-00029-s001.zip › insects-4041319-supplementary.pdf]

SUPPLEMENTAL TABLES AND FIGURES

Table S1. Characteristics of CYP genes of *P. striolata*.

Clans or subfamilies	Gene name	Gene ID	protein length	Completeness	Signal Peptide	Pfam ID	MW(kDa)	Pi
CYP2	CYP15A1	CAG9864108.1	493	Complete ORF	1-21	PF00067	57.56	7.75
CYP2	CYP18A1	CAH1186760.1	528	Complete ORF	NO	PF00067	60.86	8.26
CYP2	CYP303A1	CAG9856915.1	497	Complete ORF	1-20	PF00067	56.89	8.87
CYP2	CYP305A27	CAG9864107.1	495	Complete ORF	NO	PF00067	56.65	9.18
CYP2	CYP305A32	CAG9859303.1	500	Complete ORF	NO	PF00067	57.38	8.01
CYP2	CYP306A1	CAG9863597.1	484	Complete ORF	NO	PF00067	55.41	8.68
CYP2	CYP307A2	CAH1158455.1	393	Complete ORF	NO	PF00067	44.89	5.41
CYP2	CYP307B1	CAG9861998.1	499	Complete ORF	1-16	PF00067	56.29	8.71
CYP3	CYP345BB1	CAG9858104.1	500	Complete ORF	NO	PF00067	58.02	8.32
CYP3	CYP345BB2	CAG9858105.1	495	Complete ORF	NO	PF00067	57.51	8.86
CYP3	CYP345BJ2	CAG9858086.1	525	Complete ORF	NO	PF00067	61.27	6.53
CYP3	CYP345H7	CAG9859382.1	497	Complete ORF	NO	PF00067	57.46	9.19
CYP3	CYP345H8	CAG9859383.1	498	Complete ORF	NO	PF00067	58.02	8.18
CYP3	CYP347AC5	CAG9858620.1	496	Complete ORF	NO	PF00067	56.88	7.94
CYP3	CYP347AC6	CAG9858621.1	496	Complete ORF	NO	PF00067	57.07	7.97
CYP3	CYP3830A1	CAG9860039.1	496	Complete ORF	NO	PF00067	57.10	8.36
CYP3	CYP3832A1	CAG9863531.1	505	Complete ORF	NO	PF00067	58.04	7.78

CYP3	CYP6BQ60	CAG9859764.1	511	Complete ORF	NO	PF00067	59.26	8.8
CYP3	CYP6BQ61	CAG9859151.1	515	Complete ORF	NO	PF00067	59.51	7.14
CYP3	CYP6BQ62	CAG9859765.1	515	Complete ORF	NO	PF00067	59.79	8.86
CYP3	CYP6BQ63	CAG9859767.1	518	Complete ORF	NO	PF00067	59.65	9.02
CYP3	CYP6BQ64	CAG9860876.1	518	Complete ORF	NO	PF00067	59.93	8.93
CYP3	CYP6HD7	CAG9859827.1	511	Complete ORF	NO	PF00067	60.04	8.57
CYP3	CYP6SH4	CAG9856087.1	509	Complete ORF	NO	PF00067	58.86	8.42
CYP3	CYP6SW5	CAG9859769.1	513	Complete ORF	NO	PF00067	58.65	9.17
CYP3	CYP6SW6	CAG9859768.1	515	Complete ORF	NO	PF00067	59.64	8.65
CYP3	CYP6TF1	CAG9860120.1	510	Complete ORF	NO	PF00067	58.86	9.13
CYP3	CYP6TF2	CAH1181322.1	514	Complete ORF	NO	PF00067	59.93	8.71
CYP3	CYP6TF3	CAG9860123.1	505	Complete ORF	NO	PF00067	58.09	9.49
CYP3	CYP6TG1	CAG9857619.1	500	Complete ORF	NO	PF00067	57.19	8.26
CYP3	CYP6TH1	CAG9858842.1	497	Complete ORF	NO	PF00067	57.27	7.02
CYP3	CYP6TH2	CAG9858843.1	498	Complete ORF	NO	PF00067	57.37	9
CYP3	CYP6TJ1	CAG9858344.1	499	Complete ORF	NO	PF00067	56.89	8.03
CYP3	CYP6TJ2	CAG9858343.1	499	Complete ORF	NO	PF00067	56.93	9.1
CYP3	CYP6TJ3	CAG9858345.1	500	Complete ORF	NO	PF00067	57.29	8.79
CYP3	CYP6TK1	CAG9863637.1	505	Complete ORF	NO	PF00067	59.36	8.7
CYP3	CYP9EM1	CAG9854551.1	517	Complete ORF	NO	PF00067	59.47	9.31
CYP3	CYP9Y10	CAG9859947.1	524	Complete ORF	NO	PF00067	60.86	9.16
CYP3	CYP9Z158	CAH1185429.1	528	Complete ORF	NO	PF00067	60.88	7.28
CYP3	CYP9Z159	CAG9860586.1	525	Complete ORF	NO	PF00067	60.81	6.44
CYP3	CYP9Z160	CAG9858554.1	530	Complete ORF	NO	PF00067	61.09	6.07
CYP3	CYP9Z161	CAG9860276.1	531	Complete ORF	NO	PF00067	60.79	8.51

CYP3	CYP6SK2	CAG9854393.1	507	Complete ORF	NO	PF00067	58.67	8.8
CYP3	CYP345F13	CAG9864387.1	519	Complete ORF	NO	PF00067	60.41	8.34
CYP3	CYP6SW18	CAG9859766.1	515	Complete ORF	NO	PF00067	59.66	8.63
CYP3	CYP9Z188	CAG9858208.1	530	Complete ORF	NO	PF00067	61.29	6.85
CYP3	CYP6ZT1	CAG9859907.1	511	Complete ORF	NO	PF00067	59.36	8.53
CYP3	CYP9Z192	CAG9860809.1	512	Complete ORF	NO	PF00067	59.01	6.51
CYP3	CYP6TH3	CAH1168644.1	539	Complete ORF	NO	PF00067	62.06	8.94
CYP3	CYP6ZU1	CAG9865478.1	502	Complete ORF	NO	PF00067	58.00	8.39
CYP3	CYP6TV24	CAG9859763.1	516	Complete ORF	NO	PF00067	59.50	8.4
CYP3	CYP9Z189	CAG9865043.1	529	Complete ORF	NO	PF00067	60.94	5.78
CYP3	CYP9Z191	CAG9854600.1	527	Complete ORF	NO	PF00067	60.60	6.55
CYP3	CYP9Z190	CAG9860585.1	525	Complete ORF	NO	PF00067	61.14	6.02
CYP3	CYP6BS15	CAH1164438.1	462	Complete ORF	NO	PF00067	52.79	6.38
CYP3	CYP6YM11	CAG9858346.1	500	Complete ORF	NO	PF00067	57.72	6.81
CYP3	CYP345BB3	CAG9858106.1	492	Complete ORF	NO	PF00067	57.13	8.76
CYP3	CYP6YM9	CAG9858348.1	498	Complete ORF	NO	PF00067	57.19	8.31
CYP3	CYP9EF2	CAG9860683.1	526	Complete ORF	NO	PF00067	62.07	8.25
CYP3	CYP6SW16	CAG9860871.1	495	Complete ORF	NO	PF00067	56.74	6.14
CYP3	CYP6TJ4	CAG9858342.1	499	Complete ORF	NO	PF00067	56.89	8.87
CYP4	CYP349S1	CAG9861617.1	514	Complete ORF	NO	PF00067	59.68	9.07
CYP4	CYP3831A1	CAG9865473.1	497	Complete ORF	NO	PF00067	57.23	7.66
CYP4	CYP3831B1	CAG9858211.1	490	Complete ORF	NO	PF00067	57.58	8.42
CYP4	CYP4BN55	CAG9854240.1	495	Complete ORF	NO	PF00067	57.08	8.42
CYP4	CYP4BN56	CAG9854522.1	502	Complete ORF	NO	PF00067	58.09	8.22
CYP4	CYP4BR15	CAG9855800.1	504	Complete ORF	NO	PF00067	58.17	8.59

CYP4	CYP4G250	CAG9861388.1	559	Complete ORF	NO	PF00067	63.83	8.12
CYP4	CYP4G251	CAG9859773.1	560	Complete ORF	NO	PF00067	63.53	9.01
CYP4	CYP4NQ10	CAG9854205.1	498	Complete ORF	1-21	PF00067	57.95	9.09
CYP4	CYP4NQ9	CAG9854569.1	520	Complete ORF	NO	PF00067	60.07	9
CYP4	CYP4NQ20	CAG9854206.1	498	Complete ORF	NO	PF00067	57.74	7.57
CYP4	CYP4Q58	CAG9864834.1	498	Complete ORF	NO	PF00067	57.48	8.25
CYP4	CYP4Q59	CAG9864835.1	489	Complete ORF	1-21	PF00067	57.23	7.34
CYP4	CYP4Q60	CAG9858724.1	496	Complete ORF	NO	PF00067	57.55	7.61
CYP4	CYP4Q61	CAG9864836.1	484	Complete ORF	NO	PF00067	55.69	8.27
CYP4	CYP4Q62	CAH1168151.1	543	Complete ORF	NO	PF00067	63.03	8.34
CYP4	CYP4ST1	CAH1187334.1	495	Complete ORF	NO	PF00067	57.55	8.63
CYP4	CYP4SU1	CAG9859774.1	490	Complete ORF	NO	PF00067	57.74	9.63
CYP4	CYP4SV1	CAG9860044.1	495	Complete ORF	1-16	PF00067	56.76	8.61
CYP4	CYP4SW1	CAH1161249.1	500	Complete ORF	NO	PF00067	58.29	8.99
CYP4	CYP349F16	CAG9861488.1	526	Complete ORF	NO	PF00067	60.63	8.71
CYP4	CYP4XQ3	CAG9855799.1	488	Complete ORF	NO	PF00067	56.45	6.93
CYP4	CYP4AA11	CAG9854029.1	469	Complete ORF	NO	PF00067	54.51	9.06
CYP4	CYP349Y2	CAG9857629.1	501	Complete ORF	NO	PF00067	57.42	7.56
CYP4	CYP349S3	CAG9861616.1	527	Complete ORF	NO	PF00067	60.98	9
CYP4	CYP411C4	CAH1185633.1	486	Complete ORF	NO	PF00067	56.77	8.85
CYP4	CYP434K4	CAG9863871.1	512	Complete ORF	NO	PF00067	59.46	8.6
Mito	CYP302A1	CAG9859105.1	501	Complete ORF	NO	PF00067	58.24	10.09
Mito	CYP314A1	CAG9859221.1	490	Complete ORF	NO	PF00067	55.41	5.72
Mito	CYP315A1	CAG9865509.1	461	Complete ORF	NO	PF00067	52.91	8.53
Mito	CYP334H7	CAG9858257.1	566	Complete ORF	NO	PF00067	65.01	8.67

Mito	CYP353A10	CAG9857711.1	458	Complete ORF	NO	PF00067	52.15	9.4
Mito	CYP49A1	CAG9860317.1	544	Complete ORF	NO	PF00067	63.63	8.88

Note: *Phyllotreta striolata* genome version (PGI_PHYEVI_v3; Accession: GCA_918026865.1)

Table S2. Number of *P. striolata* full-length CYP families, subfamilies, and genes in each insect CYP clan.

clan	family	Subfamily	gene
CYP2	6	6	8
CYP3	6	26	53
CYP4	5	19	27
Mitochondrial	6	6	6
Total	23	57	94

Table S3. Primers used in this study.

Gene	Forward	Reverse
qPCR-*CYP6TG1*	GTCTACGTGGCGGATTTTCC	CATCTTCATCTTGCCCGACG
qPCR-*CYP6TH1*	AATCAAACAATGCGCCGAC	GGCTGCGTCTGGATCTTTA
qPCR-*CYP6TH2*	TCTCGGTGTTTCCTCTAGCT	GTTTTGCTTCCTGTACTCCA
qPCR-*CYP6TJ2*	CGTTCCACAAATGAAGCCGA	ATGTGCCTGTCGGTGAAGTA
qPCR-*CYP6TJ3*	CCAACTGTATCCCGACCTGT	CGCGTCCATTAACATCTGCA
qPCR-*CYP4Q62*	AGAAAATGCGGGAATGGAGC	TTTCGTCTCGTGTGCCATTG
β-actin	CCGGGAAATGCTGGAAAACA	CTGGAATGTTAGACGCTGCC
Full-*CYP6TH1*	ACGTGAGGATTGCAGTAAG	GACTGTATGTAGCTCAAGT
Full-*CYP6TH2*	AGCGACTTAAAGTAGAATAATGT	TTAATCGTTTCGTTTCAAGA
CYP6TH1-RNAi	taatacgactcactataggg-CGAATTGGCCAAAAGGAAAGA	taatacgactcactataggg-TGGGGCTCAAGGAAAATTCG
CYP6TH2-RNAi	taatacgactcactataggg-CTTTTAATTTCTTCGTGGCTGGT	taatacgactcactataggg-AGAACTGCCACTCCAAGTTT
GFP-RNAi	taatacgactcactataggg-AATGCTTTTCCCGTTATCC	taatacgactcactataggg-TGTGGTCACGCTTTTCG
CYP6TH1-28a	CGGgaattcATGCTGTCATTGGATACATTT	CGGaagtccATTTTCAACTTTTTTTAACCTAAT
CYP6TH2-28a	CGGgaattcATGTTAACTGTAGACGCAACTG	CGGaagtccTTGCTCAATTTTTTTTAATCTC

Table S4 The mortality statistics for *P. striolata* under various pyridaben treatments.

concentration(μg/mL)	mortality
100	1.19±1.19%
125	18.75±6.25%
166.7	29.27±8.00%
250	43.48±9.32%
500	54.67±5.81%

Figure S1. Expression patterns of CYP genes in *P. striolata* adults under pyridaben treatment for 24 hours at LC_{50} and LC_{10} concentrations, based on FPKM values. Treatments include a control group and groups exposed to LC_{10} or LC_{50} pyridaben (indicated below the heatmap). Red and white colors denote upregulation and downregulation, respectively.

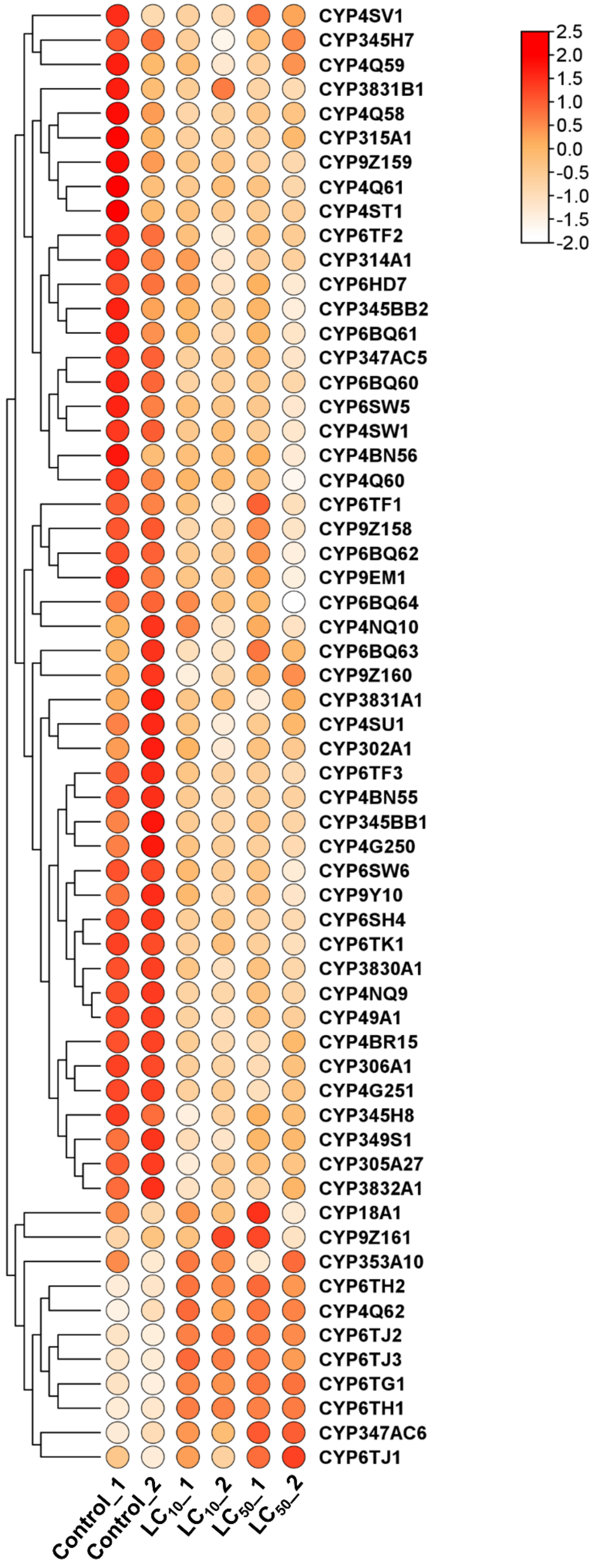

CYP4SV1
CYP345H7
CYP4Q59
CYP3831B1
CYP4Q58
CYP315A1
CYP9Z159
CYP4Q61
CYP4ST1
CYP6TF2
CYP314A1
CYP6HD7
CYP345BB2
CYP6BQ61
CYP347AC5
CYP6BQ60
CYP6SW5
CYP4SW1
CYP4BN56
CYP4Q60
CYP6TF1
CYP9Z158
CYP6BQ62
CYP9EM1
CYP6BQ64
CYP4NQ10
CYP6BQ63
CYP9Z160
CYP3831A1
CYP4SU1
CYP302A1
CYP6TF3
CYP4BN55
CYP345BB1
CYP4G250
CYP6SW6
CYP9Y10
CYP6SH4
CYP6TK1
CYP3830A1
CYP4NQ9
CYP49A1
CYP4BR15
CYP306A1
CYP4G251
CYP345H8
CYP349S1
CYP305A27
CYP3832A1
CYP18A1
CYP9Z161
CYP353A10
CYP6TH2
CYP4Q62
CYP6TJ2
CYP6TJ3
CYP6TG1
CYP6TH1
CYP347AC6
CYP6TJ1
Control_1
Control_2
LC_{10}_1
LC_{10}_2
LC_{50}_1
LC_{50}_2
2.5
2.0
1.5
1.0
0.5
0.0
-0.5
-1.0
-1.5
-2.0
